# ESPClust: unsupervised identification of modifiers for the effect size profile in omics association studies

**DOI:** 10.1093/bioinformatics/btaf065

**Published:** 2025-02-06

**Authors:** Francisco J Pérez-Reche, Nathan J Cheetham, Ruth C E Bowyer, Ellen J Thompson, Francesca Tettamanzi, Cristina Menni, Claire J Steves

**Affiliations:** School of Natural and Computing Sciences, University of Aberdeen, Aberdeen AB24 3UE, United Kingdom; Department of Twin Research and Genetic Epidemiology, School of Life Course & Population Sciences, King’s College London, London SE1 7EH, United Kingdom; Department of Twin Research and Genetic Epidemiology, School of Life Course & Population Sciences, King’s College London, London SE1 7EH, United Kingdom; Department of Twin Research and Genetic Epidemiology, School of Life Course & Population Sciences, King’s College London, London SE1 7EH, United Kingdom; The Alan Turing Institute, British Library, London NW1 2DB, United Kingdom; School of Psychology, Faculty of Science, Engineering and Medicine, University of Sussex, Brighton BN1 9QH, United Kingdom; Department of Twin Research and Genetic Epidemiology, School of Life Course & Population Sciences, King’s College London, London SE1 7EH, United Kingdom; Department of Twin Research and Genetic Epidemiology, School of Life Course & Population Sciences, King’s College London, London SE1 7EH, United Kingdom; Department of Twin Research and Genetic Epidemiology, School of Life Course & Population Sciences, King’s College London, London SE1 7EH, United Kingdom

## Abstract

**Motivation:**

High-throughput omics technologies have revolutionized the identification of associations between individual traits and underlying biological characteristics, but still use ‘one effect-size fits all’ approaches. While covariates are often used, their potential as effect modifiers often remains unexplored.

**Results:**

We propose ESPClust, a novel unsupervised method designed to identify covariates that modify the effect size of associations between sets of omics variables and outcomes. By extending the concept of moderators to encompass multiple exposures, ESPClust analyses the effect size profile (ESP) to identify regions in covariate space with different ESP, enabling the discovery of subpopulations with distinct associations. Applying ESPClust to synthetic data, insulin resistance and COVID-19 symptom manifestation, we demonstrate its versatility and ability to uncover nuanced effect size modifications that traditional analyses may overlook. By integrating information from multiple exposures, ESPClust identifies effect size modifiers in datasets that are too small for traditional univariate stratified analyses. This method provides a robust framework for understanding complex omics data and holds promise for personalised medicine.

**Availability and implementation:**

The source code ESPClust is available at https://github.com/fjpreche/ESPClust.git. It can be installed via Python package repositories as ‘pip install ESPClust==1.1.0’.

## 1 Introduction

Rapidly expanding high-throughput technologies offer an unprecedented ability to identify associations between observed traits of individuals and biological endpoints via characterisation of various ‘omics’ data. ‘Omics’ represents a range of disciplines including genomics, proteomics, and metabolomics and allows elucidation of mechanisms and processes underpinning health and disease states (Zierer *et al.* 2015, Misra *et al.* 2018, Suhre *et al.* 2021, Uffelmann *et al.* 2021, Julkunen *et al.* 2023).

Associations between omics variables and a given phenotypic outcome are often influenced by various covariates—characteristics of individuals or study conditions that may influence the observed association between an exposure (such as a biological measure) and an outcome (such as the presence of a disease). In association studies, effect size quantifies the strength of this relationship, reflecting the practical impact that changes in an exposure may have on the outcome (Kirk 2005, Ellis 2010). Covariates, which can include demographics (such as age, sex, body mass index—BMI—or ethnicity), biological diversity within samples (Gough *et al.* 2017) or technical variation in the timing of collection, collection method, and processing of samples (Leek *et al.* 2010), can affect the effect size. It is therefore standard practice to account for the potential confounding effects of such covariates when analysing associations between omics variables and outcomes.

In association studies, it is essential to distinguish between two primary roles of covariates: as confounders or as effect modifiers (or moderators). Confounders are covariates that influence both the exposure and the outcome, potentially leading to a spurious or misleading association if not accounted for. For example, age could confound an association between a gene expression level and a disease outcome if age affects both the gene expression and the risk of the disease (Steves *et al.* 2012). To isolate the true association between the exposure and outcome, it is standard practice to adjust for confounding (Lash *et al.* 2021).

Covariates can also act as effect modifiers, altering the effect size of associations rather than simply confounding them (Lash *et al.* 2021). For instance, a biomarker might show a stronger association with a health outcome in older individuals than in younger ones, indicating age as an effect modifier. However, identifying effect modifiers is not systematically considered in omics association studies, likely due to the limited statistical power of typical datasets. Neglecting effect modification not only risks skewing effect size estimates but also disregards vital heterogeneity linked to covariates, which reflects the diversity within human populations. Recognising such diversity can prove invaluable in identifying target subpopulations for maximising intervention effectiveness or delineating thresholds that differentiate groups based on distinct characteristics. Understanding how effects are different within different subpopulations is the cornerstone of developing personalised medicine.

Here, we present ESPClust, an unsupervised method to identify covariates that modify the effect size of the association between a set of omics variables and an outcome, which can be used in relatively small sample sizes for discovery science. To facilitate this, we define an effect size profile (ESP), which is a collection of effect sizes representing the connections between various omics variables and the outcome. Rather than examining each effect size in isolation as done in classical univariate analysis (Pepe *et al.* 1999, Uffelmann *et al.* 2021, Julkunen *et al.* 2023), the ESP captures the joint profile of effect sizes for multiple exposures. ESPClust works by dividing the space of covariates into regions of approximately homogeneous ESP, thus identifying clusters of individuals who share similar associations between their omics profile and the outcome. In essence, ESPClust extends the concept of effect modification to consider modification of the ESP, thereby capturing how the joint effect sizes of multiple exposures are influenced by covariates.

ESPClust is versatile and can be readily employed to explore connections between different omics datasets and outcomes. We evaluated its performance on synthetic datasets with known ground truth and applied it to real-world data to address specific research questions. The research questions were chosen to illustrate the ability of the method to (a) make discoveries in a highly researched area and (b) identify completely novel findings in an emerging disease. For the former, we investigated the relation between blood metabolomics and insulin resistance, an area which has already been heavily researched (Roberts *et al.* 2014, Pedersen *et al.* 2016, Gu *et al.* 2020), providing a valuable context for our new findings. For the latter, we explored whether and how pre-pandemic blood metabolomics, reflecting pre-infection metabolism, influenced whether someone would become symptomatic after SARS-CoV-2 infection. Although numerous studies have analysed potential relationships between COVID-19 and blood metabolomics, they often concentrate on blood samples collected post-infection or use infection severity (such as hospitalisation) as the primary outcome (Hasan *et al.* 2021, Julkunen *et al.* 2021, Marín-Corral *et al.* 2021, Sindelar *et al.* 2021, Ceballos *et al.* 2022). By considering a new question, we showcase the ability of ESPClust to analyse previously unexplored associations.

## 2 Materials and methods

### 2.1 The ESPClust method

**Figure 1.**
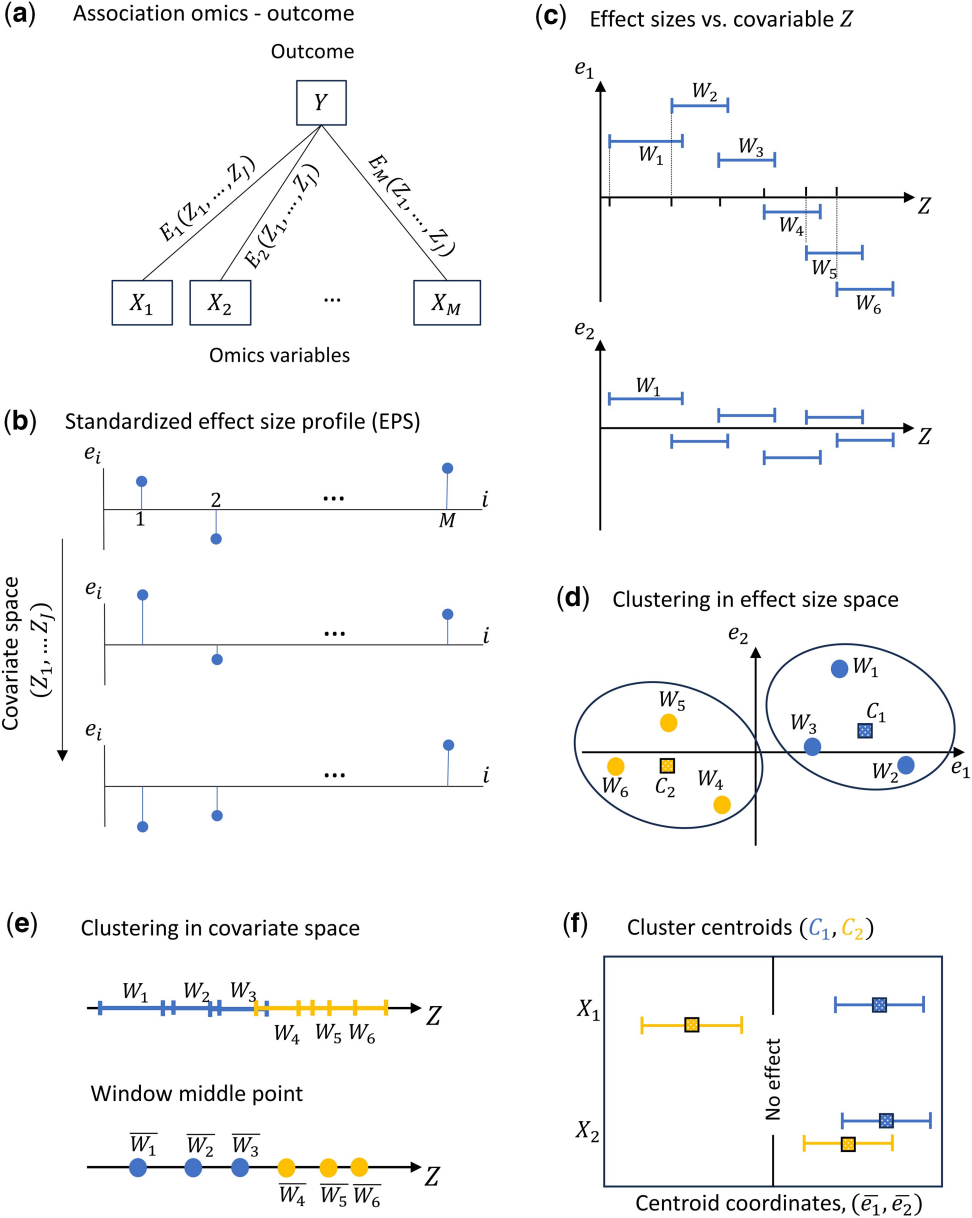
ESPClust method to identify regions in the covariate space with similar effect size profile. (a) Association between M omics variables (exposures) and an outcome Y in terms of pair-wise effect sizes $\left\{E_{1},E_{2},\ldots ,E_{M}\right\}\;$that may depend on J covariates $\left\{Z_{1},\ldots ,Z_{J}\right\}$. (b) Schematic representation of the dependence of the standardized ESP $\left\{e_{1},e_{2},\ldots ,e_{M}\right\}$ on the covariates. Panels (c)–(f) illustrate the method for a simple case with two omics variables, $\left\{X_{1},X_{2}\right\}$, which depend on a single continuous covariate, Z. (c) The effect size for each omics variable is calculated within 6 windows, ${\left\{W_{l}\right\}}_{l=1}^{6}\;$that cover the values taken by the covariate Z. (d) Clustering of the windows in the effect size space. Windows within a cluster have a similar effect size profile. $C_{1}$ and $C_{2}$ are the cluster centroids. (e) Window clusters in the covariate space defining regions shown with segments (top) or window midpoints (bottom). (f) Coordinates of the cluster centroids summarising the effect of the covariate z on the association profile of each omics variable with the outcome.

Given a set of M omics variables $\left\{X_{1},X_{2},\ldots ,X_{M}\right\}$, we define the effect size profile (ESP) as the set $\left\{E_{1},E_{2},\ldots ,E_{M}\right\}$, where each $E_{m}$ represents the association between an individual omics variable $X_{m}$ and an outcome Y (Fig. 1a). Effect sizes $\left\{E_{1},E_{2},\ldots ,E_{M}\right\}$ can be estimated through various methods depending on the research context (Kirk 2005, Ellis 2010). The method can be applied to any set of effect sizes. However, for our applications, we calculate these using coefficients from linear regression models or odds ratios from logistic regression models. The relationship between Y and $X_{m}$, adjusted for confounders $\left\{Z_{1}^{\prime },Z_{2}^{\prime },\ldots ,Z_{J\prime }^{\prime }\right\}$, is modelled as:

(1) $$f\left(Y\right)=\beta_{0}+\beta_{m}X_{m}+\sum_{j=1}^{J\prime }\alpha_{j}Z_{j}^{\prime }+\epsilon ,$$

where ϵ is a normally distributed error. For a continuous outcome, we use linear regression with $f\left(Y\right)=Y$, giving $E_{m}=\beta_{m}$. For a binary outcome, we use logistic regression with $f\left(Y\right)=\mathrm{logit}(Y)$, yielding $E_{m}=e^{\beta_{m}}$.

ESPClust standardizes the ESP to a dimensionless set $\left\{e_{1},e_{2},\ldots ,e_{M}\right\}$, derived from $\left\{E_{1},E_{2},\ldots ,E_{M}\right\}$ by subtracting the mean of each variable and normalizing by its standard deviation. We then suppose that each normalized effect size $e_{m}$ in the ESP can depend on J covariates, $\left\{Z_{1},Z_{2},\ldots ,Z_{J}\right\}$, which act as effect modifiers. The sets of confounders $\left\{Z_{1}^{\prime },Z_{2}^{\prime },\ldots ,Z_{J\prime }^{\prime }\right\}$ and effect modifiers $\left\{Z_{1},Z_{2},\ldots ,Z_{J}\right\}$ may overlap but are not necessarily identical. ESPClust generalizes univariate effect modification (Bovbjerg and Johnson 2020, Lash *et al.* 2021) to study how the entire ESP, $\left\{e_{1},e_{2},\ldots ,e_{M}\right\}$, varies across the covariate space defined by $\left\{Z_{1},Z_{2},\ldots ,Z_{J}\right\}$ (Fig. 1b).

The main aim of ESPClust is to identify regions in the covariate space where the ESP can be considered homogeneous. Within such regions, analyses that assume homogeneous effect sizes are valid. In contrast, assuming homogeneity for the ESP across distinct regions would not be justified.

ESPClust consists of three steps, outlined below.

#### 2.1.1 Step 1. Evaluation of the ESP as a function of the potential effect modifiers

Accounting for the effect modification of a discrete covariate Z such as sex or smoking status, simply requires estimating the ESP separately for each value of Z. However, with continuous covariates, the estimation process requires a more nuanced approach. Consider a scenario with J continuous covariates, $\left\{Z_{1},Z_{2},\ldots ,Z_{J}\right\}$. Directly estimating the ESP at arbitrary points in the space is often infeasible due to data sparsity.

To address this challenge, we evaluate the ESP within a set of $N_{w}$ windows $\left\{W_{1},W_{2},\ldots ,W_{N_{w}}\right\}$ that collectively cover the region of the covariate space spanned by the data. These windows, which can overlap, allow data sparsity to be handled. The concept of a ‘cover’ consisting of overlapping windows, rooted in topology (Gamelin and Greene 1999), expands upon the traditional disjoint partitioning used in stratified analysis. Overlapping windows offer advantages: they eliminate the arbitrariness in defining strata that may intersect regions with heterogeneous ESPs, such as conventional age groups. They also provide a more detailed description of the dependence of effect sizes on covariates.

**Figure 2.**
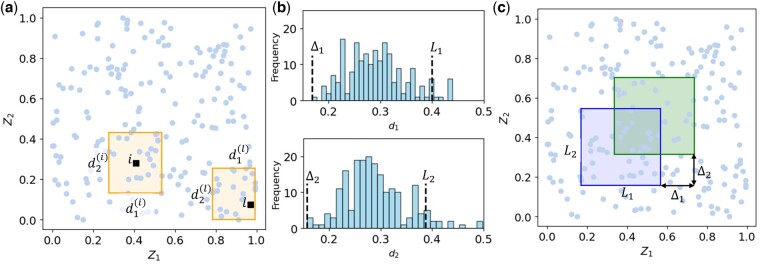
Illustration of the method for determining gliding window dimensions and step sizes in a dataset with two covariates $\left\{Z_{1},Z_{2}\right\}$. (a) Covariate space with N=200 observations (circles). For each observation, the smallest rectangle encompassing the observation and its n−1 nearest neighbours (n=20) is identified. Two example rectangles corresponding to observations i and l are shown. (b) Histograms of rectangle side lengths ($d_{1}$ and $d_{2}$) in the two covariate dimensions. The window size in each dimension is defined as the 95th percentile $\left(L_{1},L_{2}\right)$, while the gliding step $\left(\Delta_{1},\Delta_{2}\right)$ is the minimum observed side length in each dimension. (c) Example of a gliding window with dimensions $\left(L_{1},L_{2}\right)$, along with a copy shifted diagonally by $\left(\Delta_{1},\Delta_{2}\right)$.

For a single covariate (J=1), windows are one-dimensional segments (Fig. 1c). For two covariates, they are rectangles (Fig. 2c). More generally, windows correspond to J-dimensional hyperrectangles when J covariates are considered.

Once the set of windows is defined, ESPClust estimates the ESP within each window using the observations it contains and a model appropriate to the data (e.g. regression). For a given window $W_{l}$, the ESP is the set of univariate effect sizes, $\mathrm{ES}P_{l}=\{e_{1}\left(W_{l}\right),e_{2}\left(W_{l}\right),\ldots ,e_{M}\left(W_{l}\right)\}$. Step 1 of ESPClust produces a collection of $N_{W}$ effect size profiles, $\left\{\mathrm{ES}P_{1},\mathrm{ES}P_{2},\ldots ,\mathrm{ES}P_{N_{w}}\right\}$, one for each window. These profiles are then used for clustering in Step 2, as described below.

We cover the J-dimensional covariate space using a gliding hyperrectangle with dimensions $\left(L_{1},L_{2},\ldots ,L_{J}\right)$. The hyperrectangle shifts incrementally in one or more directions at a time, with step sizes $\left(\Delta_{1},\Delta_{2},\ldots ,\Delta_{J}\right)$. For instance, in two dimensions (J=2), the windows may shift horizontally by $\left(\Delta_{1},0\right)$, vertically by $(0,\Delta_{2}$), or diagonally by $\left(\Delta_{1},\Delta_{2}\right)$. Figure 2c illustrates a rectangular window in two-dimensional covariate space, along with a copy obtained through a diagonal shift.

Ideally, windows should offer a detailed description of the dependence of ESP on covariates across a wide region of the covariate space while maintaining statistically robust estimates within each window. It is crucial that each window contains enough observations to support robust ESP estimates is critical. To achieve this, we developed an automated method to determine suitable window dimensions and gliding steps, ensuring that each window typically contains at least a pre-specified number of observations, n. Figure 2 illustrates the method for two covariates.

Given a dataset with N observations and J covariates, the method standardizes all covariates and identifies the smallest hyperrectangle containing each observation and its n−1 nearest neighbours (Fig. 2a). Once the neighbourhood is determined, we back-transform to non-standardized covariates to obtain N side-length estimates for each covariate, $d_{j}^{\left(1\right)},d_{j}^{\left(2\right)},\ldots ,d_{j}^{\left(N\right)}$, where $d_{j}^{\left(i\right)}$ represents the (non-standardized) length of the jth side of the hyperrectangle for the ith observation. The jth side of the gliding windows is defined as the 95th percentile, $L_{j}=P_{95}(d_{j})$, ensuring the window is typically large enough to include at least n observations, except in sparse regions (Fig. 2b).

The gliding step in the jth direction is defined as the smallest side length of the hyperrectangles for the jth covariate, i.e. $\Delta_{j}=\min\{d_{j}^{\left(1\right)},d_{j}^{\left(2\right)},\ldots ,d_{j}^{\left(N\right)}\}$ (Fig. 2b). This definition ensures that the gliding step is finely tuned to the data distribution, allowing for a systematic exploration of the covariate space to effectively capture local heterogeneities in effect sizes.

Once the gliding steps have been calculated, the total number of windows covering the covariate space is given by $N_{w}^{\prime }=\prod_{j=1}^{J}{(n}_{j}+1)$, where $n_{j}$ is the number of steps of the gliding window in the j-th covariate, $n_{j}=\left\lfloor (Z_{j}^{\max}-Z_{j}^{\min})/\Delta_{j}\right\rfloor$. Here, $Z_{j}^{\min}$ and $Z_{j}^{\max}$ are the maximum and minimum values of the j-th covariate in the dataset, respectively, and $\left\lfloor \cdot \right\rfloor$ is the floor function. The windows are centred at $\left(Z_{1}^{\min}+i_{1}\Delta_{1},Z_{2}^{\min}+i_{2}\Delta_{2},\ldots ,Z_{J}^{\min}+i_{J}\Delta_{J}\right)$, where $i_{j}=0,1,\ldots ,n_{j}$ for j=1,2,…,J. Some of these windows may contain fewer than n observations, particularly in sparse regions of the covariate space. Accordingly, the final number of windows within which the ESP is calculated is $N_{w}\leq N_{w}^{\prime }$.

#### 2.1.2 Step 2. Clustering of windows with similar ESP

The $\mathrm{ES}P_{l}$ for each window $W_{l}$ is used as a vector of features that allows windows to be clustered in groups with similar ESP. Figure 1d illustrates the concept: Windows $W_{1},W_{2}$ and $W_{3}$ used in Fig. 1c form a cluster where both $e_{1}$ and $e_{2}$ show a positive association across all three windows. In contrast, windows $W_{4},W_{5}$ and $W_{6}$ form a separate cluster where the values of $e_{1}$ are negative and those of $e_{2}$ are positive. Agglomerative clustering will be used throughout the article, but ESPClust can be used with any other clustering method (Gan *et al.* 2007).

The number of clusters identified by ESPClust can be manually set to any positive integer. However, we implemented an automated two-step process to determine a suitable number of clusters:

- Determine the most frequent optimal cluster number $k_{1}$ across four clustering indices: Calinski-Harabasz (Calinski and Harabasz 1974), Davies-Bouldin (Davies and Bouldin 1979), silhouette (Rousseeuw 1987), and elbow (Willmott 2019). If there is no repeated number across the four indices or in the case of a tie, $k_{1}$ is set to the smallest value among all the scores. The Calinski-Harabasz, Davies-Bouldin, and silhouette methods determine the optimal number of clusters by maximising their respective scores. The elbow method identifies the optimal number of clusters by locating the value k for which the change in slope of the clustering quadratic error (inertia, $I_{k}$) is maximal. This corresponds to the most prominent ‘elbow’ in the inertia vs. k plot. The slope of the inertia is defined as $s_{k}=I_{k}-I_{k-1}$, and the relative change of the slope as $\delta_{k}=\frac{s_{k}}{s_{k+1}}-1$. The optimal k corresponds to the maximum of $\delta_{k}$.
- The clustering indices of step (i) split the windows into more than one group (i.e. $k_{1}>1$) even when the effect size is homogeneous over the covariate space. To test the statistical significance of splitting the data into $k_{1}>1$ clusters compared to a non-clustered null hypothesis (k=1), we generate $n_{\mathrm{ref}}$ reference datasets, with each feature uniformly distributed within the observed range of values. For each reference dataset, r, the slope change $\delta_{k_{1}}^{r}$ is computed. A p-value is then calculated as the proportion of reference datasets where $\delta_{k_{1}}^{r}>\delta_{k_{1}}$, indicating cases where the observed elbow at $k_{1}$ is no more prominent than in the reference data. The null hypothesis of one cluster is rejected in favour of k>1 if the p-value is below a significance level α.

#### 2.1.3 Step 3. Identification of regions in the covariate space with homogeneous ESP

This step uses the window clusters obtained in Step 2 to identify regions in the covariate space with homogeneous ESP. Figure 1e illustrates the process for a case with a single effect modifier. In this example, the covariate space is split into two regions with ‘small’ and ‘large’ Z. Since the windows covering the covariate space can overlap, hard clustering of windows in the effect size space results in fuzzy clustering in the covariate space (Gan *et al.* 2007). For instance, in Fig. 1e, segments overlap, allowing a specific value of the modifier to belong to multiple windows simultaneously. Figure 2c exemplifies the overlap of windows in a two-dimensional covariate space. This approach acknowledges that individuals sharing a common modifier (e.g. age) may exhibit varied associations between omics variables and outcomes. Nonetheless, we employ the midpoint of each window, $\bar{W_{l}},$ to provide a clearer visualization of regions characterized by homogeneous ESP (see the lower panel in Fig. 1e).

The centroids of the clusters serve as summaries of the ESP within each region, aiding in the interpretation of how covariates influence the relationship between each omics variable and the outcome. In Fig. 1f, the coordinates of the centroids and their dispersion are displayed. In this instance, the influence of the covariate Z on the association between the pair $\left(X_{1},Y\right)$ is significantly greater than that on the pair $\left(X_{2},Y\right)$. This is evident from the larger difference between the centroids $C_{1}$ and $C_{2}$ for $X_{1}$compared to those for $X_{2}.$ Ultimately, this discrepancy reflects the stronger dependency of $e_{1}$ on Z, as illustrated in Fig. 1c.

### 2.2 Synthetic data

To evaluate the performance of ESPClust, we applied it to synthetic datasets with a known ground truth.

**Figure 3.**
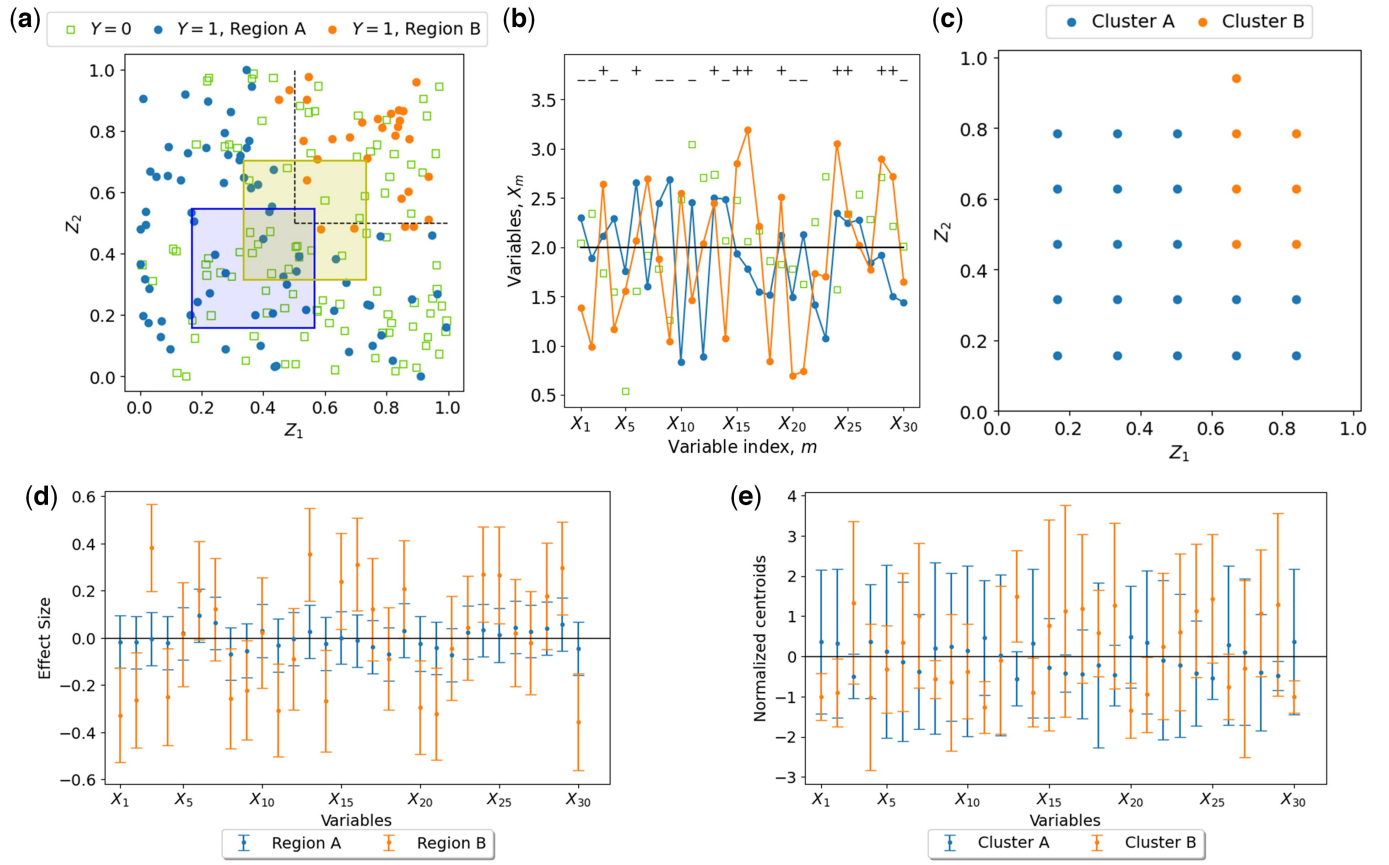
Application of ESPClust to synthetic data of type D2 with a dichotomous outcome, Y∈{0,1}, and two effect modifiers, $\left\{Z_{1},Z_{2}\right\}$. (a) Observations in the covariate space, with symbol shapes and colours denoting outcome (Y) and region (A and B) as indicated in the legend. Dashed lines separate regions with distinct associations. Two example windows are shown, each of size $\left(L_{1},L_{2}\right)=(0.4,0.4)$ and shifted diagonally by $\left(\Delta_{1},\Delta_{2}\right)=(0.16,0.16)$. (b) Omics variables for three selected observations with differing outcomes and/or association type (see legend in panel (a)). Symbols ‘+’ and ‘–’ mark omics variables with ${\bar{X}}_{m}=2.7$ and ${\bar{X}}_{m}=1.3$ for observations with Y=1 in region B. (c) Clusters in the covariate space identified by ESPClust. Symbols indicate the centres of windows. (d) Effect size for omics variables for each region in the covariate space. (e) Coordinates of the cluster centroids. Error bars in (d) and (e) represent 1.96σ, where σ is the standard deviation of centroid coordinates.

All datasets consider a dichotomous outcome Y with values {0,1} and consist of 100 observations for each class. Each observation includes 30 omics variables, $\left\{X_{1},X_{2},\ldots ,X_{30}\right\}$, and two effect modifiers, $\left\{Z_{1},Z_{2}\right\}$. The values of $Z_{1}$ and $Z_{2}$ were drawn uniformly from [0,1] (Fig. 2a). Three types of datasets were built which assume different dependences of the omics-outcome associations on the covariates $\left\{Z_{1},Z_{2}\right\}$:

1. *Datasets D1*: These datasets model situations without effect modification such that the effect sizes $\left\{e_{1},e_{2},\ldots ,e_{30}\right\}$ do not depend on $\left\{Z_{1},Z_{2}\right\}$. This is achieved by drawing the omics variables from a normal distribution with mean ${\bar{X}}_{m}=2$ and standard deviation 0.5 for all m=1,2,…,30 (i.e. $X_{m}∼N({2,0.5}^{2})$). This distribution was used for observations with both Y=0 and Y=1, ensuring no dependency on $Z_{1}$ or $Z_{2}$.
2. *Datasets D2*: The covariate space $\left\{Z_{1},Z_{2}\right\}$ is divided into *two regions* with distinct associations between the omics variables and the outcome: Region A, where either $Z_{1}$ or $Z_{2}\leq 0.5$, and region B, where $Z_{1},Z_{2}>0.5$. To make the regions more realistic, the boundary between them was smoothed, creating a gradual transition in membership probabilities near the thresholds (see dashed lines in Fig. 3a). For observations with Y=0, the omics variables were generated independently of $\left\{Z_{1},Z_{2}\right\}$. These variables were drawn as in D1 ($X_{m}∼N({2,0.5}^{2})$). These observations are shown as empty squares in Fig. 3a and b. For observations with Y=1:
  - In region A, the omics variables followed the same distribution as Y=0, indicating no statistical association between the omics variables and the outcome in this region (orange circles in Fig. 3a and b).
  - In region B, the 30 omics variables were divided into three mutually exclusive random sets, each containing 10 variables. Within each set, the omics variables were drawn from $X_{m}∼N({\bar{X}}_{m},0.5^{2})$, with ${\bar{X}}_{m}=2,2.7$ and 1.3 for the three sets, respectively. This setup reflects the possibility that only a subset of the omics variables are influenced by the effect modifiers $Z_{1}$ and $Z_{2}$.
3. *Datasets D3*: The covariate space $\left\{Z_{1},Z_{2}\right\}$ is divided into *three regions* with distinct associations between the omics variables and the outcome: Region A, where $Z_{1}<0.5$, region B, where $Z_{1},Z_{2}>0.5,$ and region C, where $Z_{1}>0.5$ and $Z_{2}<0.5$. The omics variables for Y=0 were generated as in datasets D2 ($X_{m}∼N({2,0.5}^{2})$). This same distribution was also used for observations with Y=1 in region A. In region B the omics variables were constructed similarly to region B in datasets D2. In region C, the variables were drawn as an independent random sample, following the same distribution as in region B but assigned to new random sets of 10 variables each. This reflects the presence of differential associations between the omics variables and the outcome in Regions B and C, driven by the effect modifiers.

### 2.3 Insulin resistance data

We used ESPClust to investigate the impact of BMI, sex, and gut microbiome gene richness (i.e. the number of unique microbial genes) on the relationship between serum metabolites and insulin resistance (HOMA-IR) among 275 non-diabetic individuals from the Danish MetaHIT study (Metagenomics of the Human Intestinal Tract | METAHIT | Project | Fact sheet | FP7 | CORDIS | European Commission) (see Supplementary Table S1). Results will be presented for two examples using different metabolomic variables as exposures, as described in (Lynch and Pedersen 2016, Pedersen *et al.* 2018). We restricted our analysis to known metabolites within these datasets.

### 2.4 COVID-19 symptoms data

We utilized ESPClust to explore the potential of BMI, sex, and age as modifiers for the association between metabolomic variables collected before COVID-19 infection and the manifestation of COVID-19 symptoms (i.e. symptomatic or asymptomatic).

#### 2.4.1 Study population

Participants were selected from the UK Adult Twin Registry (King's College London) based on the following criteria: they had pre-pandemic metabolomic data available, information on COVID-19 symptom status, and evidence of SARS-CoV-2 infection.

#### 2.4.2 Exposure variables

The metabolite concentrations of fasting blood samples collected before the COVID-19 pandemic were measured with two different platforms that yielded the two metabolic datasets used in this study (see Supplementary Table S2).

The first dataset was obtained through a high-throughput nuclear magnetic resonance (NMR) platform (Soininen *et al.* 2009, Würtz *et al.* 2017) by Nightingale Health Ltd, Helsinki, Finland. This platform provides the concentration of over 200 circulating metabolic biomarkers including lipids, fatty acids, amino acids, ketone bodies glycolysis-related metabolites as well as lipoprotein subclass distribution and particle size. Specifically, the first dataset consists of 221 biomarkers obtained from the serum of 680 participants of the TwinsUK cohort study(TwinsUK—The biggest twin registry in the UK for the study of ageing-related diseases).

The second dataset (C19-1) was obtained using an untargeted liquid chromatography-mass spectrometry (LC-MS) procedure conducted by Metabolon, Inc., Durham, North Carolina, USA as previously described (Evans *et al.* 2014, Shin *et al.* 2014). Our dataset comprises 774 pre-pandemic serum metabolites obtained from 368 TwinsUK participants.

#### 2.4.3 Outcome variable

The outcome variable giving the symptom status was derived from self-reported symptoms (Bowyer *et al.* 2023) in TwinsUK COVID-19 questionnaires (Suthahar *et al.* 2021), administered between July 2020 and February 2022, and serology data (Cheetham *et al.* 2023). Infected participants were classified into two groups: asymptomatic or symptomatic. Participants were labelled as asymptomatic if they reported that had not had COVID-19 but there was evidence of SARS-CoV-2 infection. In contrast, participants were assumed to be symptomatic if there was evidence of natural infection, reported having had COVID-19 and provided the duration of their symptoms (this was imposed to strengthen the evidence that these patients were symptomatic). Information on symptoms was obtained from three TwinsUK COVID-19 questionnaires (Suthahar *et al.* 2021) administered in July–August 2020 (Q2), October–November 2020 (Q3), and November 2021–February 2022 (Q4). Evidence of SARS-CoV-2 infection was confirmed through antibody testing conducted around the times of Q2 and Q4. Testing data were supplemented with self-reported vaccination records to ensure accuracy; participants were considered infected if they had a positive anti-Nucleocapsid result at any time or a positive anti-Spike result prior to vaccination (Interim Guidelines for COVID-19 Antibody Testing | CDC).

#### 2.4.4 Missing data and data transformation

Metabolites whose concentration was missing for more than 20% of individuals were discarded. Similarly, individuals who missed more than 20% of the metabolites were also discarded. The remaining missing values for metabolites were imputed using k Nearest Neighbours (Mendez *et al.* 2019) with k=3.

Sex was encoded as a numerical variable (0 for male and 1 for female); the remaining variables are intrinsically numerical. Metabolites were individually transformed by adding one and applying the natural logarithm function. All variables were individually standardized by subtracting the mean value and dividing by the standard deviation.

## 3 Results

### 3.1 Synthetic data

To gain statistical insight, we applied ESPClust to identify clusters in the covariate space $\left\{Z_{1},Z_{2}\right\}$ across 500 datasets of each type (D1–D3). Windows containing at least n=20 observations were used. While the value of n is chosen by the analyst, it is essential to select sufficiently large values when dealing with dichotomous outcomes. This ensures that both outcome values are represented within the windows, which is necessary for calculating effect sizes. Effect sizes within windows were estimated using univariate logistic regression.

Table 1 shows a contingency matrix summarising the number of inferred clusters for each type of dataset for a significance level $\alpha =10^{-3}$ in Step 2.ii of ESPClust.

**Table 1.** Contingency matrix showing the distribution of clusters identified by ESPClust across 500 datasets for each type (D1, D2, and D3).

		Number of clusters				
		1	2	3	4	5
Number of regions	1	457	13	23	7	0
	2	7	486	7	0	0
	3	22	34	394	43	7

Datasets of type D1, D2, and D3 contain 1, 2, and 3 true regions, respectively. Rows represent the number of true regions, while columns represent the number of clusters identified by the method.

Datasets D1 were designed to simulate a uniform association across the whole covariate space, where one would expect a single cluster with homogeneous association. ESPClust performed well, identifying one cluster in 457 datasets D1 (91% of cases).

For datasets D2, where two clusters corresponding to regions A and B are expected, ESPClust identified two clusters in 486 of the datasets, representing a 97% frequency. Figure 3c shows the clusters discovered by ESPClust for a dataset of type D2, which approximate the predefined regions A and B indicated in Fig. 3a. The lack of points near the boundaries of the covariate space is due to windows containing fewer than n=20 observations.

Notably, the differences in the statistical associations of individual omics variables with the outcome across regions A and B are small, as shown in Fig. 3d by the overlapping effect sizes for most metabolites. Similarly, the centroids of the two clusters show only minor statistical differences (Fig. 3e). Despite these subtle variations, ESPClust successfully identified two distinct clusters. This demonstrates its ability to leverage the ESP rather than relying solely on individual effect sizes, overcoming the limitations of traditional stratification and univariate analysis in such scenarios.

For datasets D3, where three clusters corresponding to regions A, B, and C are expected, ESPClust identified three clusters in 394 simulations (79% of cases, Table 1). While the performance is lower than for datasets D2, it remains impressive given the subtle differences between regions A, B, and C at the level of individual omics variables. Supplementary Figure S1 illustrates the results for a particular dataset of type D3.

These results demonstrate the robustness and accuracy of ESPClust in detecting association clusters across diverse datasets, even when individual-level differences in effect sizes are small or indistinguishable.

To obtain the results presented above, ESPClust calculated the window dimensions $\left(L_{1},L_{2}\right)$ and gliding steps $(\Delta_{1},\Delta_{2}$) for each dataset as described in Step 1. To assess the sensitivity of ESPClust to variations in window dimensions and gliding steps, we replicated the analysis presented above while varying them from the baseline values of $\left(L_{1},L_{2}\right)$ and $(\Delta_{1},\Delta_{2}$) used in the above analysis.

Halving the gliding steps led ESPClust to identify a single cluster in 391 out of 500 datasets of type D1 (Supplementary Table S3), a decline compared to the 457 cases identified using the baseline parameters (Table 1). Similarly, increasing the window dimensions to $\left(1.5L_{1},1.5L_{2}\right)$ while keeping the baseline gliding steps resulted in further deterioration, with only 365 datasets of type D1 correctly identified as having one cluster (Supplementary Table S5).

In contrast, increasing the gliding steps to $\left(1.5\Delta_{1},1.5\Delta_{2}\right)$ while maintaining the baseline window dimensions improved performance in terms of D1 datasets, with ESPClust identifying a single cluster in 482 datasets of type D1 (Supplementary Table S4). However, this improvement came at the expense of the ability to correctly identify two or three clusters, which deteriorated significantly compared to the baseline parameters (cf. Supplementary Table S4 and Table 1).

Overall, the baseline parameters provide a good balance, achieving robust accuracy for cases with and without effect modifiers.

### 3.2 Insulin resistance data

**Figure 4.**
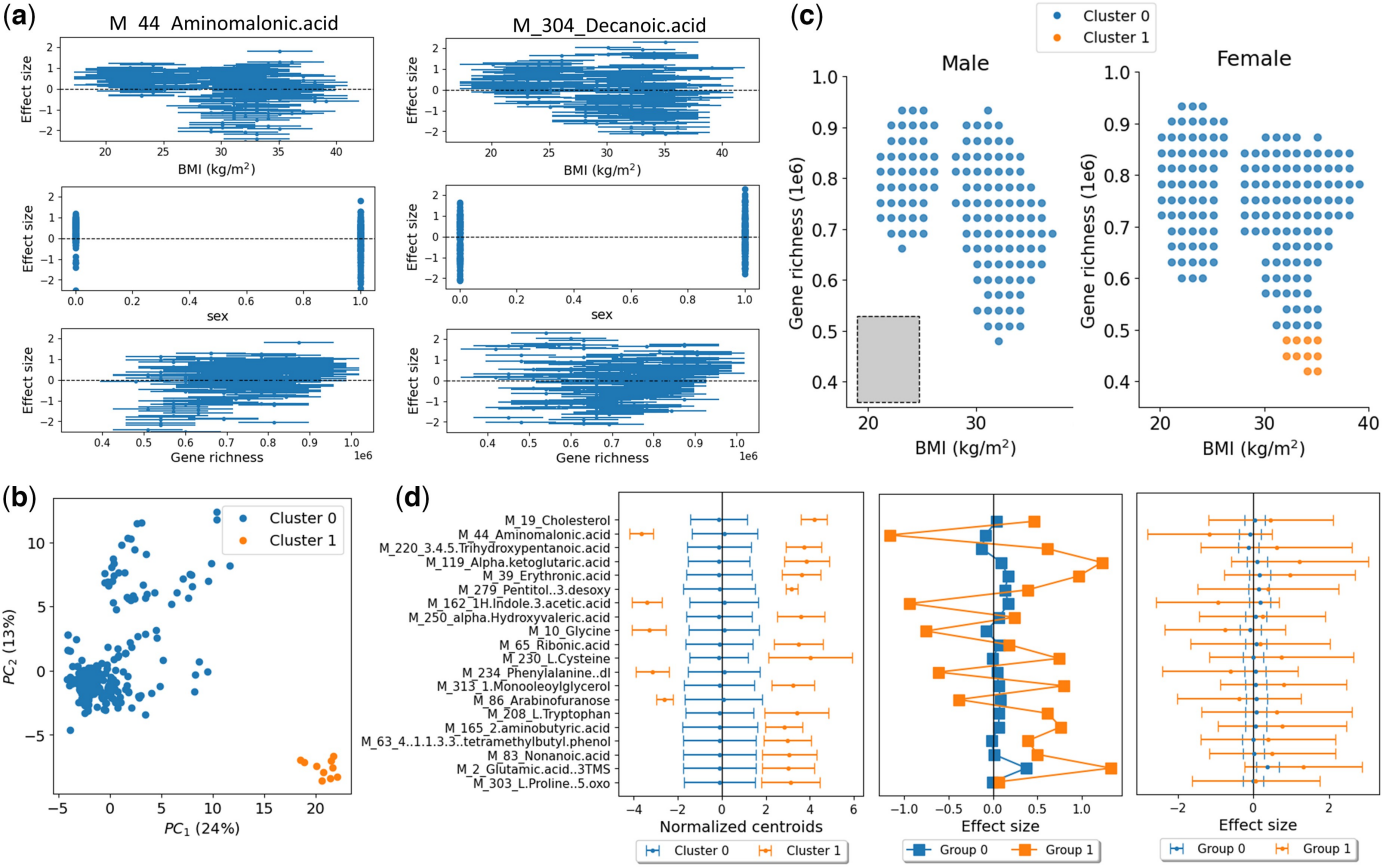
Application of ESPClust to study the association between insulin resistance and 94 serum metabolites. (a) Example of the effect size within windows for aminomalonic acid and decanoic acid in the covariate space (BMI, sex, gene richness). The error bars in the plots for BMI and gene richness indicate the size of the window used to cover these covariates. (b) Visualization of two clusters with different ESP using the first two principal components of the windows effect sizes. (c) Clusters in the covariate space separately shown for males and females. The symbols indicate the middle point of the windows used to estimate the effect sizes. The size of the window used to calculate effect sizes for fixed sex is shown by a grey rectangle. (d) The left panel shows the coordinates of the cluster centroids corresponding to the 20 metabolites that differ the most between clusters. The error bars indicate 1.96σ, where σ is the standard deviation of the centroid coordinates. The central panel shows the effect size for the same metabolites for two groups of individuals representing the two identified clusters. The third panel shows the same effect sizes as in the second panel but the 95% confidence intervals for the effect sizes are shown with error bars.

For the first application of ESPClust to insulin resistance data, we used 94 polar metabolites from the Danish MetaHIT study as the exposures (Pedersen *et al.* 2016). The effect sizes of step 1 in ESPClust were calculated with linear regression for each sex, using windows of size ${(L}_{\mathrm{BMI}},L_{g.\mathrm{rich}.})=(5.7\mathrm{kg}/m^{2},168\;895)$ and gliding steps $\left(\Delta_{\mathrm{BMI}},\Delta_{g.\mathrm{rich}.}\right)=(1.0\mathrm{kg}/m^{2},30\;294)$ (see grey rectangle in Fig. 4c). Only windows containing more than 10 observations (n>10) were considered. Within each window, the effect size for every metabolite was calculated as the slope of a linear regression model, adjusting for both BMI and gene richness.

Figure 4a shows the dependence of the effect size on each of the covariates for two metabolites which exemplify different levels of heterogeneity: the association between aminomalonic acid and insulin resistance exhibits more pronounced variation with the covariates compared to that of decanoic acid.

ESPClust splits the windows into two clusters (*P*-value $<10^{-3}$). This also looks appropriate when projecting the window effect sizes onto the two first principal components (Fig. 4b). Figure 4c shows that Cluster 1 comprises female individuals with high BMI and low gene richness. It cannot be established whether Cluster 1 extends to the region of high BMI and low gene richness for males due to insufficient data in this region for this group.

In Fig. 4d (left), the centroid coordinates corresponding to the 20 metabolites with the most significant differences between clusters are shown. This highlights that, in addition to differing in ESP collectively, distinct clusters also diverge at the level of specific individual metabolites (such as cholesterol or glycine).

Previous studies have suggested that elevated levels of cholesterol are linked with obesity (i.e. high BMI), increased insulin resistance (Mc Auley 2020), and reduced gene richness (Cotillard *et al.* 2013). Our findings suggest that BMI and insulin resistance may not only confound the association between cholesterol and insulin resistance but also act as effect modifiers, amplifying the positive association in cluster 1 (i.e. among individuals with high BMI and low gene diversity). Similarly, ESPClust indicates that the negative association between glycine levels and insulin resistance (Alves *et al.* 2019) is moderated by BMI and gene diversity.

To compare the results of ESPClust with traditional univariate methods using stratification, we categorised individuals into two groups representing the clusters identified by ESPClust. Cluster 1 was represented by individuals with BMI ≥ 26 kg/m^2^ and gene richness ≤ 480 000 (Cotillard *et al.* 2013, Le Chatelier *et al.* 2013); cluster 0 was represented by the rest of individuals.

Figure 4d (centre) shows that the effect size for the most distinct metabolites exhibits trends similar to those of the centroid coordinates (Fig. 4d (left)). However, the differences in effect sizes do not achieve statistical significance, as evidenced by the overlap of error bars in Fig. 4d (right). This reinforces the idea illustrated above for synthetic data: ESPClust can identify clusters with varying ESP levels that may not be discernible combining traditional stratification and univariate analysis. Indeed, ESPClust may detect collective differences between effect size profiles that may not be prominent at the level of individual omics variables unless large datasets are used to enhance the power of univariate analysis.

As a second example in the context of insulin resistance, we utilised ESPClust with 289 lipids from the Danish MetaHIT study as exposures (Pedersen *et al.* 2016). Our findings mirrored those obtained with 94 metabolites. Using gliding windows of the same size and step as in the 94 metabolites dataset, the covariate space was again divided into two clusters, splitting the (BMI, gene richness) space in a similar manner. However, with the 289 lipids dataset, cluster 1—corresponding to individuals with high BMI and low gene richness—extended to higher values of gene richness and included the male subspace (Supplementary Fig. S2a).

The coordinates of the cluster centroids for the lipidomic dataset did not exhibit statistically significant differences between the clusters (Supplementary Fig. S2b). However, there is a noticeably tendency for several triglycerides (e.g. TG(56:5)) to show higher effect sizes within Cluster 1. This aligns with previous studies linking elevated triglyceride levels to obesity, low gene richness, and metabolic disorders such as insulin resistance (Le Chatelier *et al.* 2013, Agus *et al.* 2021). Our findings suggest that the positive association between triglycerides and insulin resistance is particularly strengthened in regions characterized by high BMI and low gene richness.

### 3.3 COVID-19 symptoms data

Since the symptomatic/asymptomatic outcome in the COVID-19 datasets is binary, the potential ESP dependence on BMI, age and sex was estimated through univariate logistic regression within windows that cover the covariate space.

**Figure 5.**
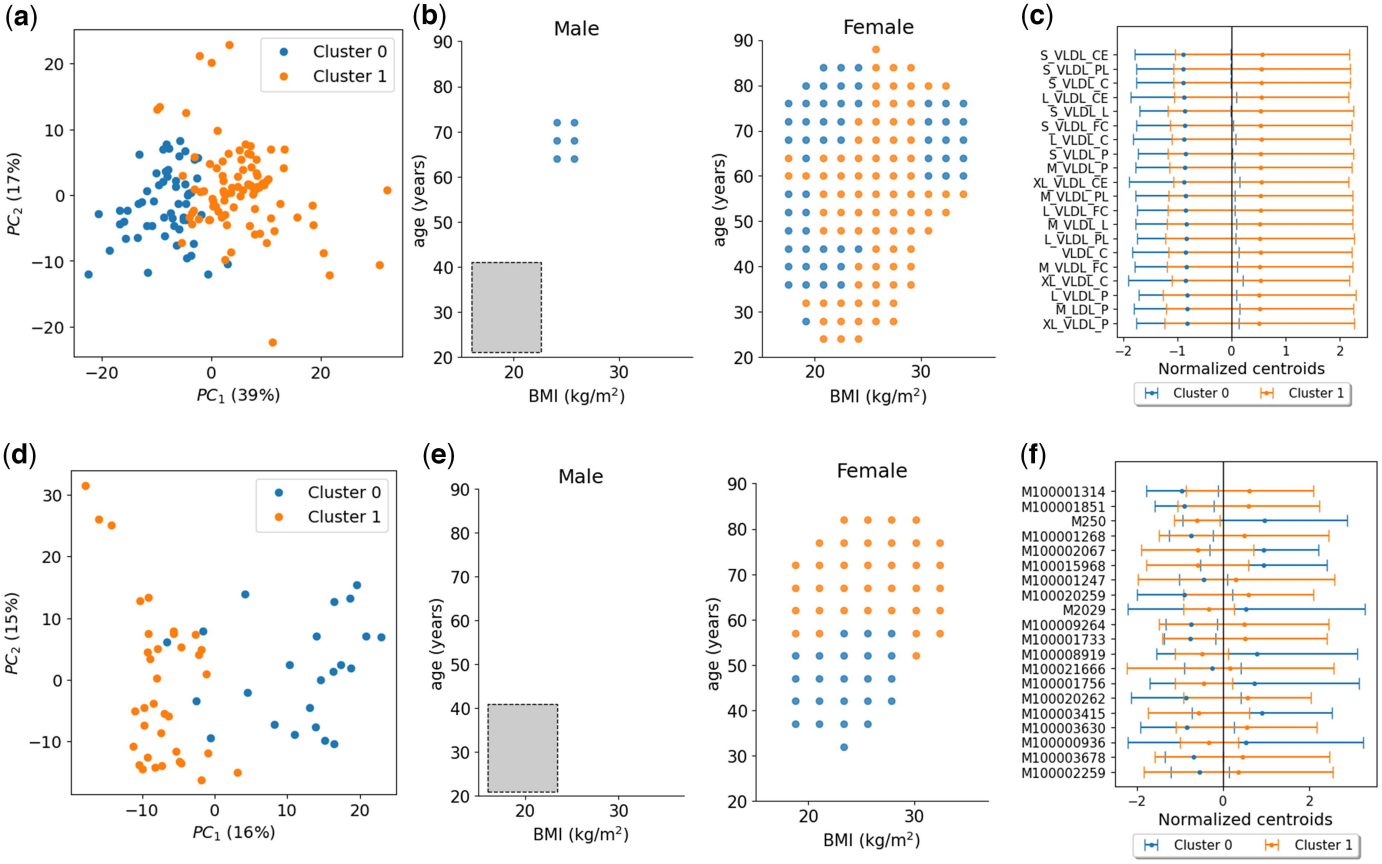
Results obtained by employing EPSClust to investigate the potential role of BMI, sex, and age on the association between COVID-19 symptoms manifestation and serum metabolomics. Panels (a–c) show findings from ESPClust analysis using 221 NMR blood biomarkers as exposures, while panels (d–f) present analogous results obtained using 774 LC-MS blood metabolites. (a, d) Visualisation of the clusters for the ESP, using the first two principal components of the window effect sizes. (b, e) Clusters in the covariate space separately shown for male and female individuals. The symbols indicate the middle point of the windows used to estimate effect sizes. The size of the gliding window used to calculate effect sizes for fixed sex is represented by a grey rectangle in the panel for male individuals. (c, f) Coordinates of the cluster centroids corresponding to the 20 metabolites that differ the most between clusters. The error bars indicate 1.96σ, where σ is the standard deviation of the centroid coordinates.

For the first dataset, based on 221 NMR metabolites, we applied ESPClust with a minimum of n=25 observations per window. This resulted in window sizes ${(L}_{\mathrm{BMI}},L_{\mathrm{age}})=(6.6\mathrm{kg}/m^{2},20\mathrm{years})$ and a gliding step of $\left(\Delta_{\mathrm{BMI}},\Delta_{\mathrm{age}}\right)=(1.7\mathrm{kg}/m^{2},4\mathrm{years})$ for each sex, which were grouped into two clusters (Fig. 5). Although the clustering seems reasonable in the PCA plot of Fig. 5a, the differences between the two clusters are not easily interpretable in terms of BMI, age, and sex.

The coordinates of the cluster centroids overlap, and no individual metabolite exhibits a distinctly different effect size across clusters (Fig. 5c). In this application, the differences between the clusters are therefore linked to the EPS as a whole. Nevertheless, there is a notable trend for several VLDL and LDL ratios to show higher effect sizes in Cluster 1. A larger dataset might lead to a statistically clearer trend in this direction.

For the second metabolomics dataset, which includes 774 LC-MS metabolites, imposing n=25 yielded window sizes of ${(L}_{\mathrm{BMI}},L_{\mathrm{age}})=(7.5\mathrm{kg}/m^{2},20\mathrm{years})$ and gliding steps of $\left(\Delta_{\mathrm{BMI}},\Delta_{\mathrm{age}}\right)=(2.3\mathrm{kg}/m^{2},5\mathrm{years})$ for each sex. ESPClust splits the windows into two clusters in the female subspace (Fig. 5e) which appear reasonable separated in the PCA plot (Fig. 5d). No windows in the male subspace contained sufficient data to estimate the ESP (Fig. 5e).

In terms of age, the split of windows in the female subspace obtained using the 774 LC-MS metabolites (Fig. 5e, right) is more intuitive than that obtained using 221 NMR metabolites (Fig. 5b, right). A possible explanation for the enhanced interpretability is that increasing the number of metabolites enhances the resolution of the dependence of the EPS on the covariates. At the level of individual metabolites, however, significant overlap between clusters persists (Fig. 5f).

## 4 Discussion

We have introduced ESPClust, a flexible method for unsupervised identification of effect size modifiers in omics association studies. The method expands upon the concept of effect size modification, traditionally related to the association between an exposure-outcome pair, to utilise the information provided by a set of effect sizes for the association of multiple omics variables and an outcome. This collection of effect sizes defines the effect size profile, referred to as ESP. ESPClust finds regions in the covariate space with differing ESP, effectively generalising the effect size modification concept for individual omics variables to the concept of ESP modification.

An ESP modifier may function as an effect modifier for individual omics variables, as demonstrated in our analysis of serum metabolites and insulin resistance. However, ESP modification captures phenomena not discernible at the individual omics level, as shown in our synthetic data or the COVID-19 symptom phenotype analysis.

A crucial step in ESPClust is to decide if there is more than one region in the covariate space with differing ESP, i.e. whether there is evidence supporting the split of ESP windows into more than one cluster. ESPClust uses a test that compares the slope changes of the clustering inertia in the observed data with the changes expected for non-clustered data. We explored the possibility of using the gap statistic instead which is often used to test if the data falls into a single cluster (Tibshirani *et al.* 2001). However, the gap statistic systematically grows with the number of clusters when applied to our synthetic data and does not allow us to identify a single cluster even for datasets of type D1 with homogeneous association across the whole covariate space (Supplementary Fig. S3).

ESPClust offers considerable potential for advancing personalised medicine by identifying subpopulations with distinct biological responses. By detecting covariate-specific effect size modifications even using relatively small datasets, ESPClust reveals subtle heterogeneities that traditional methods may miss. This ability to tailor interventions based on individual biological profiles can enhance treatment efficacy and precision. Consequently, ESPClust facilitates the development of more personalised healthcare strategies, improving patient outcomes and driving progress in the field of personalised medicine.

Although this paper has focused on omics association studies, ESPClust is very versatile and can be applied well beyond this field. Indeed, it can be applied to any problem involving features used to predict outcomes influenced by effect modifiers, as it excels at identifying context-dependent relationships and clustering features based on shared effect size patterns. By accounting for how moderators shape associations, ESPClust provides a framework to uncover nuanced, subgroup-specific insights that are crucial for understanding complex systems and tailoring interventions across diverse domains.

## Supplementary Material

btaf065_Supplementary_Data

## Data Availability

The metabolomic data for the examples of insulin resistance are available at (Pedersen *et al.* 2018) https://bitbucket.org/hellekp/clinical-micro-meta-integration/src/master/. The data used for the COVID-19 examples are held by the Department of Twin Research at King’s College London. The data can be released to bona fide researchers using our normal procedures overseen by the Wellcome Trust and its guidelines as part of our core funding (https://twinsuk.ac.uk/resources-for-researchers/access-our-data/).
