# Supplementary material for: ESPClust: unsupervised identification of modifiers for the effect size profile in omics association studies
Source: Bioinformatics. 2025 Feb 6;41(2):btaf065. doi: 10.1093/bioinformatics/btaf065 (PMC11879214; doi:10.1093/bioinformatics/btaf065)
Supplement: btaf065_Supplementary_Data [file btaf065_supplementary_data.docx]

**Supplementary Tables**

Table S1. Demographics relevant to the application of ESPClust to the association between serum metabolomics and insulin resistance. The statistics for insulin resistance, BMI, and gene richness are given as Median (2.5% percentile, 97.5% percentile).

| **sex** | **n** | **Insulin resistance** | **BMI (kg/m^2^)** | **Gene richness** |
| --- | --- | --- | --- | --- |
| Male | 125 | 1.8 (0.4,4.9) | 30.9 (20.4,39.9) | 744470 (407391,1013525) |
| Female | 150 | 1.5 (0.5,5.4) | 30.7 (19.6,41.5) | 736092 (378970,958634) |
| All | 275 | 1.7 (0.4,5.2) | 30.7 (20.0,41.2) | 743324 (398365,984865) |

Table S2. Demographics relevant to the application of ESPClust to the association between serum metabolomics and COVID-19 symptoms manifestation. The statistics for BMI and age are given as Median (2.5% percentile, 97.5% percentile)

| **Omics data** | **Symptoms class** | **Sex (M/F)** | **n** | **BMI (kg/m^2^)** | **Age (years)** |
| --- | --- | --- | --- | --- | --- |
| 221 NMR biomarkers | Asymptomatic | Male | 53 | 24.5 (19.9,34.4) | 65 (24,86) |
|  |  | Female | 292 | 25.4 (18.4,38.9) | 67 (31,84) |
|  | Symptomatic | Male | 34 | 25.4 (20.3,33.5) | 57 (25,74) |
|  |  | Female | 301 | 24.5 (18.2,37.5) | 56 (25,77) |
| 774 LC-MS metabolites | Asymptomatic | Male | 31 | 24.8 (18.9,33.1) | 69 (33,87) |
|  |  | Female | 153 | 25.3 (18.8,36.0) | 67 (34,84) |
|  | Symptomatic | Male | 19 | 25.6 (21.7,30.3) | 59 (31,77) |
|  |  | Female | 165 | 25.3 (18.4,36.0) | 56 (31,76) |

Table S3. Contingency matrix showing the distribution of clusters identified by ESPClust across 500 datasets for each type (D1, D2, and D3). Results obtained using windows with dimensions $(L_{1}, L_{2})$ and gliding steps $({0.5\Delta}_{1},{0.5\Delta}_{2})$, where $\left( L_{1}, L_{2} \right)$ and $(\Delta_{1}, \Delta_{2})$ are the baseline parameters defined in step 1 of ESPClust. Datasets of type D1, D2, and D3 contain 1, 2, and 3 true regions, respectively. Rows represent the number of true regions, while columns represent the number of clusters identified by the method.

|  | | Number of clusters | | | | |
| --- | --- | --- | --- | --- | --- | --- |
|  |  | 1 | 2 | 3 | 4 | 5 |
| Number of regions | 1 | 391 | 51 | 41 | 17 | 0 |
|  | 2 | 2 | 495 | 3 | 0 | 0 |
|  | 3 | 7 | 31 | 418 | 43 | 1 |

Table S4. Contingency matrix showing the distribution of clusters identified by ESPClust across 500 datasets for each type (D1, D2, and D3). Results obtained using windows with dimensions $(L_{1}, L_{2})$ and gliding steps $({1.5\Delta}_{1},{1.5\Delta}_{2})$, where $\left( L_{1}, L_{2} \right)$ and $(\Delta_{1}, \Delta_{2})$ are the baseline parameters defined in step 1 of ESPClust. Datasets of type D1, D2, and D3 contain 1, 2, and 3 true regions, respectively. Rows represent the number of true regions, while columns represent the number of clusters identified by the method.

|  | | Number of clusters | | | | |
| --- | --- | --- | --- | --- | --- | --- |
|  |  | 1 | 2 | 3 | 4 | 5 |
| Number of regions | 1 | 482 | 9 | 7 | 2 | 0 |
|  | 2 | 29 | 457 | 13 | 1 | 0 |
|  | 3 | 78 | 30 | 304 | 78 | 10 |

Table S5. Contingency matrix showing the distribution of clusters identified by ESPClust across 500 datasets for each type (D1, D2, and D3). Results obtained using windows with dimensions $({1.5L}_{1}, 1.5L_{2})$ and gliding steps $(\Delta_{1},\Delta_{2})$, where $\left( L_{1}, L_{2} \right)$ and $(\Delta_{1}, \Delta_{2})$ are the baseline parameters defined in step 1 of ESPClust. Datasets of type D1, D2, and D3 contain 1, 2, and 3 true regions, respectively. Rows represent the number of true regions, while columns represent the number of clusters identified by the method.

|  | | Number of clusters | | | | |
| --- | --- | --- | --- | --- | --- | --- |
|  |  | 1 | 2 | 3 | 4 | 5 |
| Number of regions | 1 | 365 | 41 | 56 | 38 | 0 |
|  | 2 | 2 | 495 | 3 | 0 | 0 |
|  | 3 | 20 | 25 | 388 | 63 | 4 |

**Supplementary figures**

**ESPClust: Unsupervised identification of modifiers for the effect size profile in omics association studies**


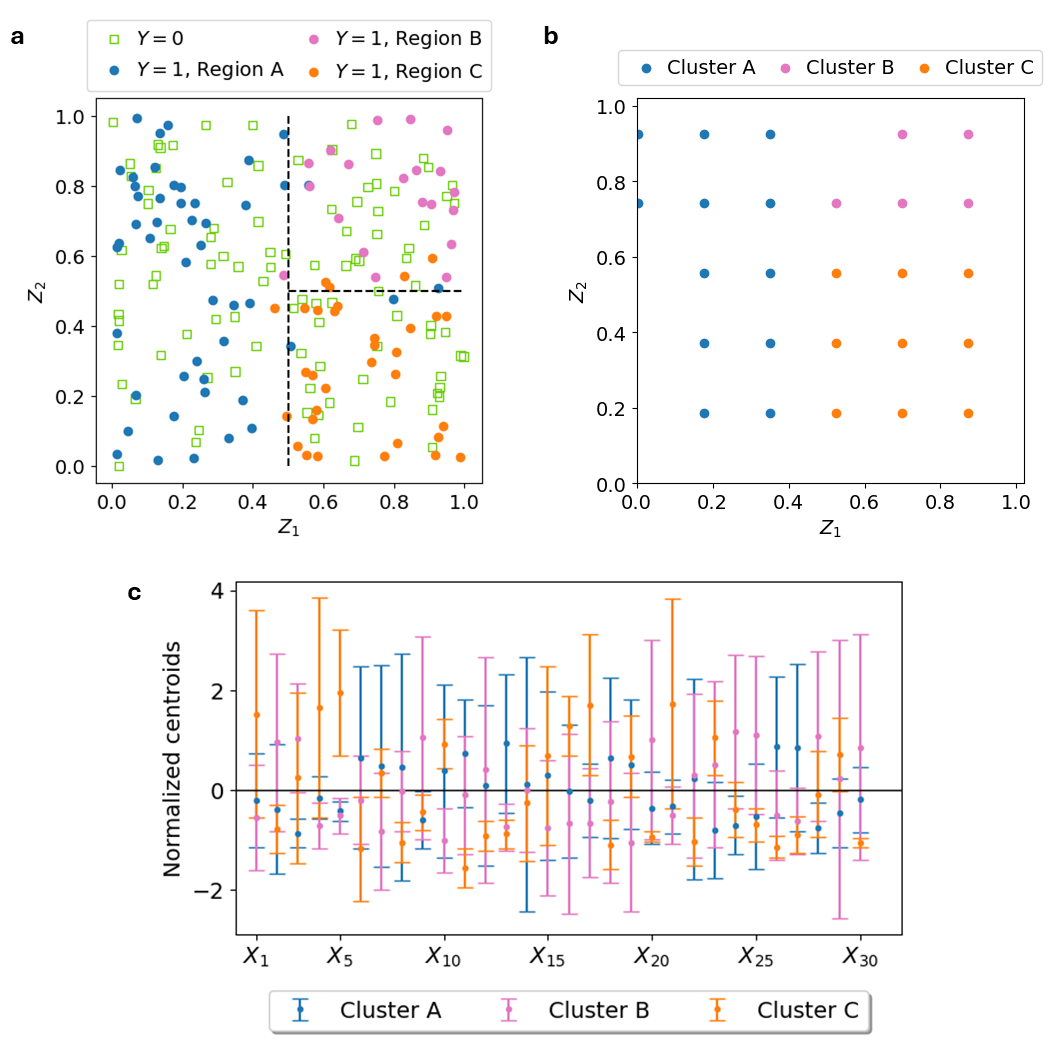


Figure S1. Application of ESPClust to synthetic data of type D3 with a dichotomous outcome, $Y\in\{0,1\}$, and two effect modifiers, $\{Z_{1},Z_{2}\}$. (a) Observations in the covariate space, with symbol shapes and colours denoting outcome ($Y$) and region (A, B and C) as indicated in the legend. Dashed lines separate regions with distinct associations. (b) Clusters in the covariate space identified by ESPClust. Symbols indicate the centres of windows. (c) Coordinates of the cluster centroids. Error bars represent $1.96\sigma$, where $\sigma$is the standard deviation of centroid coordinates.


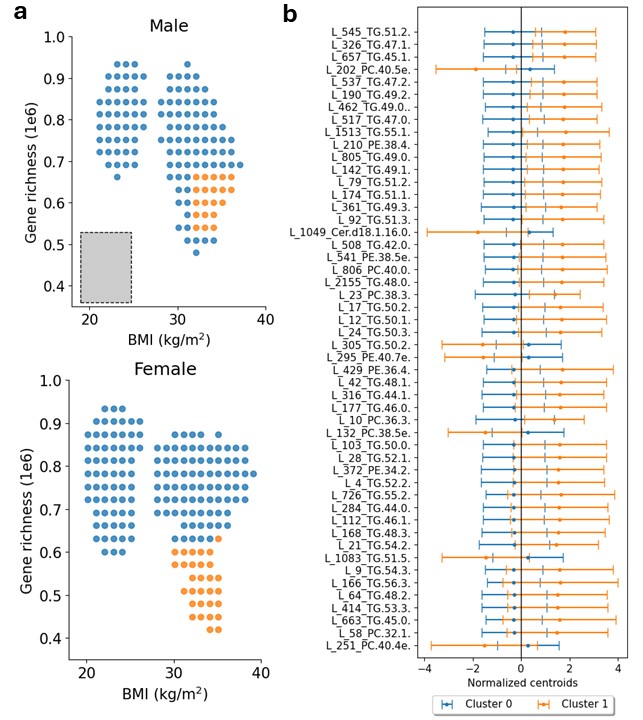


Figure S2. Application of ESPClust to study the association between insulin resistance and 289 lipids serum metabolites. (a) Clusters in the covariate space separately shown for males and females. The symbols indicate the middle point of the windows used to estimate the effect sizes. The size of the window used to calculate effect sizes for fixed sex is shown by a grey rectangle. (b) Coordinates of the cluster centroids corresponding to the 50 lipids that differ the most between clusters. The error bars indicate 1.96$\sigma$, where $\sigma$ is the standard deviation of the centroid coordinates.


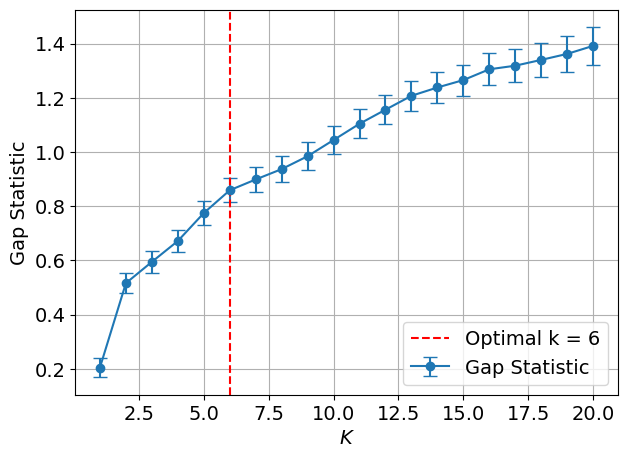


Figure S3. Application of the gap statistic to determine the number of clusters of windows in an application of ESPClust to a synthetic dataset of type D1. The gap statistic is plotted as a function of the number of clusters. The ESP is independent of the covariates in this dataset and a single cluster would be expected.
